# Nanoribbon Biosensor-Based Detection of microRNA Markers of Prostate Cancer

**DOI:** 10.3390/s23177527

**Published:** 2023-08-30

**Authors:** Yuri D. Ivanov, Kristina A. Malsagova, Kristina V. Goldaeva, Svetlana I. Kapustina, Tatyana O. Pleshakova, Vladimir P. Popov, Andrey F. Kozlov, Rafael A. Galiullin, Ivan D. Shumov, Dmitry V. Enikeev, Natalia V. Potoldykova, Vadim S. Ziborov, Oleg F. Petrov, Alexander Y. Dolgoborodov, Alexander V. Glukhov, Sergey V. Novikov, Victoria K. Grabezhova, Evgeniy S. Yushkov, Vladimir A. Konev, Oleg B. Kovalev, Alexander I. Archakov

**Affiliations:** 1 Institute of Biomedical Chemistry (IBMC), 119121 Moscow, Russia; yurii.ivanov.nata@gmail.com (Y.D.I.); kristina.malsagova86@gmail.com (K.A.M.); sveta.kapustina7.05@gmail.com (S.I.K.); topleshakova@yandex.ru (T.O.P.); afkozlow@mail.ru (A.F.K.); rafael.anvarovich@gmail.com (R.A.G.); shum230988@mail.ru (I.D.S.); alexander.archakov@ibmc.msk.ru (A.I.A.); 2 Rzhanov Institute of Semiconductor Physics, Siberian Branch of Russian Academy of Sciences, 630090 Novosibirsk, Russia; popov@isp.nsc.ru; 3 Institute for Urology and Reproductive Health, Sechenov University, 119992 Moscow, Russia; enikeev_dv@mail.ru (D.V.E.); natalis8282@mail.ru (N.V.P.); 4 Joint Institute for High Temperatures of Russian Academy of Sciences, 125412 Moscow, Russia; ziborov.vs@yandex.ru (V.S.Z.); ofpetrov@ihed.ras.ru (O.F.P.); aldol@ihed.ras.ru (A.Y.D.); 5 JSC “Novosibirsk Plant of Semiconductor Devices with OKB”, 630082 Novosibirsk, Russia; gluhov@nzpp.ru; 6 Associate Printing-and-Publication Centre Technosphera, 125319 Moscow, Russia; svnovikov59@mail.ru; 7 JSC “Design Center for Biomicroelectronic Technologies “Vega””, 630082 Novosibirsk, Russia; vikavega10@yandex.ru; 8 Department for Business Project Management, National Research Nuclear University “MEPhI”, 115409 Moscow, Russia; 9 Department of Infectious Diseases in Children, Faculty of Pediatrics, Pirogov Russian National Research Medical University, 117997 Moscow, Russia; konev60@mail.ru (V.A.K.); doctor87@list.ru (O.B.K.)

**Keywords:** prostate cancer, microRNA, silicon-on-insulator, nanoribbon, biomarker

## Abstract

Prostate cancer (PC) is one of the major causes of death among elderly men. PC is often diagnosed later in progression due to asymptomatic early stages. Early detection of PC is thus crucial for effective PC treatment. The aim of this study is the simultaneous highly sensitive detection of a palette of PC-associated microRNAs (miRNAs) in human plasma samples. With this aim, a nanoribbon biosensor system based on “silicon-on-insulator” structures (SOI-NR biosensor) has been employed. In order to provide biospecific detection of the target miRNAs, the surface of individual nanoribbons has been sensitized with DNA oligonucleotide probes (oDNA probes) complementary to the target miRNAs. The lowest concentration of nucleic acids, detectable with our biosensor, has been found to be 1.1 × 10^−17^ M. The successful detection of target miRNAs, isolated from real plasma samples of PC patients, has also been demonstrated. We believe that the development of highly sensitive nanotechnology-based biosensors for the detection of PC markers is a step towards personalized medicine.

## 1. Introduction

Prostate cancer (PC) is one of the most common types of cancer occurring in men [1], being the fifth cause of death in the world [2,3]. PC is characterized by high morbidity and mortality rates, particularly among elderly men. According to the WHO, PC ranks second in primary disease detection and sixth in morbidity rate among all oncological diseases [3,4]. Similar to other types of cancer, PC is a multifactorial disease. Its main causes include genetic and ecological factors [5]. The main features of this disease include early cancer onset (starting at the age of 30), high malignancy of primarily diagnosed cancer, and fast progression [6]. These features determine the importance of timely diagnosis of PC.

PC develops without any visible signs and complaints, often beginning to cause discomfort at only late stages. Early stages of the disease can be either completely asymptomatic, or accompanied by concomitant, more common pathologies such as benign prostatic hyperplasia, thus hindering early diagnosis of PC [7]. Currently, commonly employed methods of clinical PC diagnosis include prostatectomy in the case of localized cancer, and androgen deprivation therapy in the case of metastasis [8]. However, surgical resection is accompanied by a high risk of complications, with urinary incontinence and erectile dysfunction being the most common ones [9].

To date, the commonly available methods of instrumental diagnosis of PC are imperfect. The commonly employed methods of clinical PC diagnosis include manual and digital rectal examination, magnetic resonance tomography, and computer tomography [2,10]. Recently, multiparametric magnetic resonance imaging (mpMRI) was proposed for the diagnosis of PC [11]. Transrectal ultrasound-guided biopsy of the prostate still remains the “gold standard” in this respect [12]. This method allows one to detect cancer cells in prostate tissue, to assess the Gleason score and, thus, to determine the treatment strategy to be used by the clinician [10]. At the same time, biopsy is an invasive approach, which causes discomfort to the patient. Furthermore, systematic biopsy in the case of active monitoring of patients with clinically insignificant PC (Gleason score < 7) can overlook PC progression. The latter can lead to late PC diagnosis [13,14]. In addition, the fact that a human is involved in the evaluation of results, obtained by the above-listed methods of PC diagnosis, inevitably leads to subjectivity in the interpretation of the obtained data: the results of one and the same study can be interpreted differently if the patient is examined by more than one clinician. Importantly, the above-listed methods are macroscopic ones, and their sensitivity is insufficient for the early revelation of cancer. In this respect, nanotechnology-based methods open up the opportunity for both the early diagnosis and the effective treatment of PC [2]. In their extensive review, Barani et al. [2] emphasized that early revelation of PC can allow one to enhance the effectiveness of its treatment [2,15], improving the survival rate from 10% to 90% [2,16].

The preferable painless approaches to early diagnosis of PC include serological liquid biopsy with the use of disease-specific biochemical markers. These markers allow one to perform fast and accurate diagnosis. Prostate-specific antigen (PSA) is widely employed as a PC biomarker [2]. PSA-based screening consists of the determination of the PSA level in blood samples. Despite the wide use of PSA-based screening in practical healthcare, the proportion of patients with PC—in particular, with stage III PC—remains quite high and amounts to ~45% [17]. Furthermore, conventional PSA-based screening often leads to false positive results due to insufficient marker specificity [18]. According to the American Urological Association, the proportion of men with PSA > 3.0 ng/mL and without PC is 75.9% after follow-up biopsy [19]. PSA is known to be synthesized in the prostate [20]. Nevertheless, it is not considered as a 100% tumor-specific marker of PC, since an increase in the PSA level can be related to prostatitis, benign prostatic hyperplasia, etc. [10]. Furthermore, the molecular mechanisms of PC metastasis remain largely unknown [21]. It is thus important to determine the genetic drivers of PC in order to find new biomarkers for stratification of the risk and aggressiveness of PC during screening examinations. The PC aggressiveness depends on the degree of tumor tissue differentiation, and on the stage at which the disease is revealed. Indeed, Ferraro et al. [7] stated that currently it is insufficient to solely rely on the results of PSA-based tests. This is the reason why the search for new PC-specific biomarkers [22] is required in order to provide early PC revelation. 

Takahashi et al. justified the importance of considering ribonucleic acids (RNAs) as PC biomarkers [22]. Among these biological macromolecules, microRNAs (miRNAs) form a large family of short, highly conserved noncoding RNA molecules [23]. These RNAs were reported to regulate the expression of several target genes, which are involved in such normal biological processes as proliferation, differentiation, and apoptosis [24]. To date, ~2000 different miRNAs have been identified in humans, and their number is growing [25]. In several studies, abnormal miRNA expression in some types of cancer, in which miRNAs act as either tumor suppressors or oncogens, was reported [26,27]. Recent studies revealed the potential of some miRNAs to act as diagnostic biomarkers [27,28]. Circulating miRNAs were revealed in such biological fluids as blood, saliva, and urine. Wong et al. found miR-184 in the blood of 80% of patients with tongue squamous cell carcinoma—as compared with only 13% of healthy people [29]. In regards to PC, Shen et al. emphasized that certain miRNAs—namely, miR-20a, miR-21, miR-145, and miR-221—are associated with its development and progression [30]. Waseem et al. considered miR-183-5p as a PC biomarker and showed that miR-183 expression correlates with increased PSA level, higher Gleason score, and metastases [31]. Yung et al. identified 63 miRNAs with differential expression in the same categories of PC patients [32]. Sabahi et al. reported the use of miR-21 as a PC biomarker [33]. 

Promising methods include the detection of PC using nanotechnology-based biosensors [2,33,34,35,36,37,38]. Among them, one should single out biosensors containing miniaturized chips “silicon-on-insulator”-based nanoribbon structures (SOI-NR biosensors) [2,39,40,41,42,43,44,45,46,47]. These biosensors allow one to detect biological markers of human diseases in biological fluids at very low concentrations (<10^−15^ M [42,43,44,45,46,47]), which correspond to early stages of cancer [15]. The key feature of the SOI-NR biosensor systems is their extremely high sensitivity to charged particles owing to the small characteristic size and, hence, high surface-to-volume ratio of the sensor element [48]. With respect to biological macromolecules, this key feature of the SOI-NR biosensors allows one to achieve 10^−17^ M to 10^−15^ M detection limits. Another benefit of this type of biosensor is the label-free detection of target molecules in real time [39,40,41,42,43,44,45,46,47,49,50]. Namely, subfemtomolar detection limits were attained for the SOI-NR biosensor-based assay upon detection of protein [45,46] and miRNA [42,43,44] molecules.

In the present study, we have used a biosensor, which comprised an array of “silicon-on-insulator” (SOI) nanoribbon sensor structures. The latter had been fabricated by a complementary metal-oxide semiconductor (CMOS)-compatible technology, with the use of gas-phase reduction and lithography. In order to provide biospecific detection, the surface of the sensor structures had been sensitized by covalent immobilization of DNA oligonucleotide probes (oDNA probes). 

Herein, we demonstrate how the use of a nanoribbon array, formed on a single sensor chip, has allowed us to perform simultaneous detection of a palette of biomarkers comprising several PC-associated miRNAs by immobilizing different oligonucleotide probes on each individual nanoribbon. Nucleotide sequences of the probes were complementary to those of four target miRNAs, which were previously reported to be associated with PC (miRNA-183 [31], miRNA-346 [51], miRNA-429 [52], and miRNA-484 [53]). Our study comprised two steps. For the first step, experiments on the detection of model DNA oligonucleotides (oDNAs) in buffer solution were performed in order to determine the detection limit attainable with our biosensor. The sequences of these model oDNAs correspond to those of the target miRNAs, i.e., the model oDNAs represent synthetic analogues of the target miRNAs. For the second step of the study, we investigated whether it is possible to detect the target miRNAs isolated from the real samples of plasma of PC patients. At this step, successful detection of the target miRNAs was demonstrated.

## 2. Materials and Methods

### 2.1. Chemicals

The following chemicals were used in our experiments: isopropanol (“AcrosOrganics”, Geel, Belgium), hydrofluoric acid (“Reakhim”, Moscow, Russia), ethanol (“Reakhim”, Moscow, Russia), 3,3′-dithiobis(sulfosuccinimidyl propionate) (DTSSP) cross-linker (Pierce, Waltham, MA, USA), monocalcium phosphate (MCP, Sigma Aldrich, St. Louis, MO, USA), dimethyl sulfoxide (DMSO, Sigma Aldrich, St. Louis, MO, USA), and 3-aminopropyltriethoxysilane (APTES, Sigma Aldrich, St. Louis, MO, USA). Deionized water was obtained with a Simplicity UV purification system (Millipore, Molsheim, France).

### 2.2. Oligonucleotides

All oligonucleotides used in the experiments were synthesized by Evrogen (Moscow, Russia). The oDNA probes named “probe_1”, “probe_2”, “probe_3”, and “probe_4” were used for the sensitization of the surface of nanoribbons. Table 1 lists nucleotide sequences of the oDNA probes.

In the experiments on the determination of the detection limit, model oDNAs were used as target molecules. These model oDNAs, designated as “CS_1”, “CS_2”, “CS_3”, and “CS_4”, represent synthetic analogues of target miRNAs. Nucleotide sequences of the model oDNAs are complementary to those of oDNA probes with the same numeric designation. Table 2 lists the nucleotide sequences of the model oDNAs.

The nucleotide sequences listed in Table 1 and Table 2 were determined using a miRBase database [54]. Since mature miR-3p and miR-5p can also circulate in the blood and have various sequences, we used the sequences of immature miRNA183, miRNA 346, miRNA 429, and miRNA 484 in order to provide the detection of any mature form of the respective miRNAs.

### 2.3. Preparation of Buffered Solution of Target oDNAs

The solutions of the model oDNAs with concentrations ranging from 10^−18^ M to 10^−15^ M were prepared from the initial stock solution (100 μM in 50 mM monocalcium phosphate (MCP), pH 7.4) by tenfold serial dilution with buffer solution (1 mM MCP, pH 7.4). At each dilution step, the solution was incubated in a shaker for 30 min at 10 °C and 600 rpm. The solutions were prepared immediately before their use in the experiments.

### 2.4. Collection of Blood Plasma Samples

All samples were collected according to protocols of I.M. Sechenov First Moscow State Medical University (Sechenov University) in compliance with the order no. 1177n (Ministry of Health of Russian Federation; 20 December 2012). Blood plasma samples were obtained from patients with PC diagnosed during either medical examination or surgery. The studies were performed in accordance with the ethical committee; patients provided informed consent for participation in the study involving human biomaterial.

We analyzed plasma samples of patients with confirmed PC (No. 5 and 44). Blood plasma samples from patients with benign cyst of the left kidney (No. 27) were used as control samples. Table 3 lists the characteristics of blood plasma samples.

Blood sampling was conducted on an empty stomach from the cubital vein before treatment. Samples were collected in vacutainers with 3.8% Sodium Citrate anticoagulant (S-Monovette^®^, Sarstedt, Germany) and centrifuged at 3000 rpm for 6 min at room temperature. Each plasma sample (500 µL) was collected into two dry test tubes, frozen, and stored at −80 °C prior to its use in the experiments. MiRNAs were extracted from the plasma samples with a miRCURY RNA Isolation Kit—Biofluids immediately before the experiments.

### 2.5. Fabrication of SOI-NR Chips

**Figure 1:**
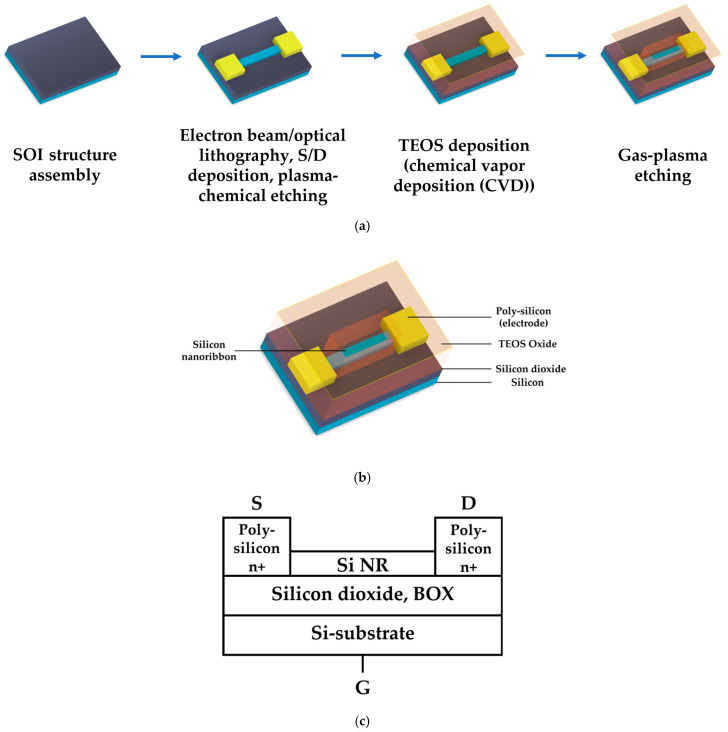
Schematic illustration of the workflow of the SOI-NR chip fabrication process, which includes SOI structure assembly, electron beam/optical lithography, source-drain (S/D) contacts deposition, plasma-chemical etching, tetraethyl orthosilicate (TEOS) deposition (chemical vapour deposition), and gas-plasma etching (**a**). The schematic images of the resulting structure (**b**) and its cross-section (**c**).

The SOI-NR chips were fabricated as described in detail elsewhere [36,45,47]. Figure 1a schematically illustrates the workflow of the chip fabrication process, while the components of the resulting SOI structure are specified in Figure 1b. 

**Figure 2:**
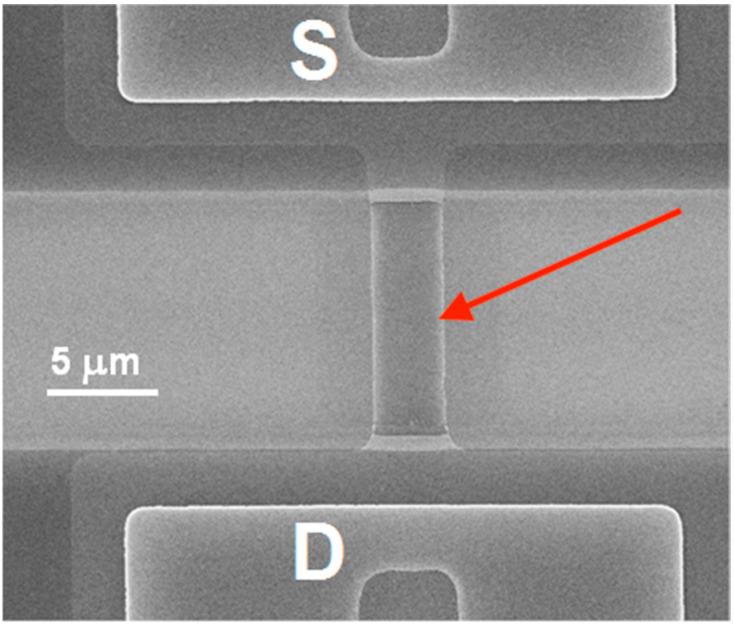
Typical SEM image of a nanoribbon. Red arrow indicates nanoribbon location between the source (S) and drain (D) contacts. The scale bar is 5 µm.

The SOI-NR chips were fabricated using electron beam lithography and gas-plasma chemical etching [46]. The drain-source regions were formed by polysilicon layer deposition followed by doping. The resulting n^+^-ohmic contacts determined the enrichment mode for n-SOI-NR structures during measurements. SOI-NR were grouped into pairs, so that each SOI-NR sensor chip comprised six pairs of nanoribbons. In order to perform measurements in electrolyte solutions, a tetraethyl orthosilicate layer was deposited onto the surface of the crystal with the nanoribbons. SOI-based nanoribbon structures had n-type conductivity. The cut-off silicon layer was 32 nm thick, while the buried oxide (BOX) layer was 300 nm thick. The nanoribbon width, thickness, and length were 3 μm, 32 nm, and 10 μm, respectively. Figure 2 displays a typical SEM image of a nanoribbon.

### 2.6. Surface Treatment of SOI-NR Chips

The SOI-NR chip surface was treated with isopropanol to remove mechanical impurities. The native oxide formed on the chip surface during storage was eliminated using hydrofluoric acid solution in ethanol. The chip was then treated in an ozone cleaner (UV Ozone Cleaner—ProCleaner™ Plus, Ossila Ltd., Sheffield, UK) in order to form hydroxyl groups on the nanoribbon surface, providing its further silanization with APTES according to the previously described protocol [55], which was developed based on the technique reported by Yamada et al. [56].

### 2.7. Sensitization of the Nanoribbons

**Figure 3:**
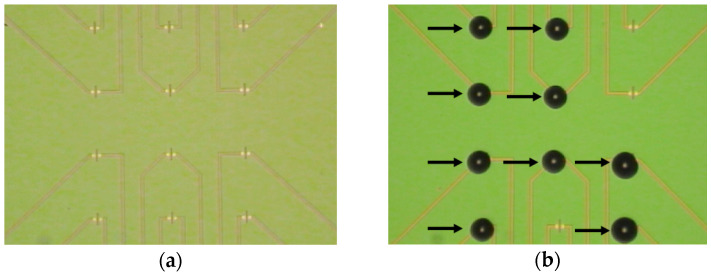
Optical images of the SOI-NR chip surface before (**a**) and after (**b**) dispensing 1 nL droplets of oDNA immobilization solutions onto the surface of individual nanoribbons. The 1 mM solutions of any of the four oDNA probes were dispensed with an iONE-600 non-contact robotic system equipped with a piezoelectric dispenser.

oDNA probes (probe_1, probe_2, probe_3, and probe_4), specified in Table 2, were covalently immobilized onto the silanized surface of nanoribbons using DTSSP cross-linker. For this purpose, nanoliter droplets of solutions containing any of the oDNA probes at a concentration of 1 μM in MCP (50 mM, pH 7.4) were precisely dispensed onto the DTSSP-activated surface of individual nanoribbons with a non-contact robotic iONE-600 liquid handling system equipped with a piezoelectric dispenser (M2-Automation GmbH, Berlin, Germany). The volume of the probe oDNA solution dispensed onto each individual nanoribbon was typically ~1 nL. Figure 3 displays optical images of the SOI-NR chip surface before (Figure 3a) and after (Figure 3b) dispensing the probe oDNA solutions onto the nanoribbons.

After dispensing the solutions of the oDNA probes on the surface of the nanoribbons, the SOI-NR chip was incubated for a long time (24 h) in a humid chamber. Then, the SOI-NR chip surface was washed with deionized water. In biosensor experiments, nanoribbons sensitized with oDNA probes were used as working sensors, while those without immobilized oDNA probes on the surface were used as control sensors.

### 2.8. SOI-NR Biosensor

**Figure 4:**
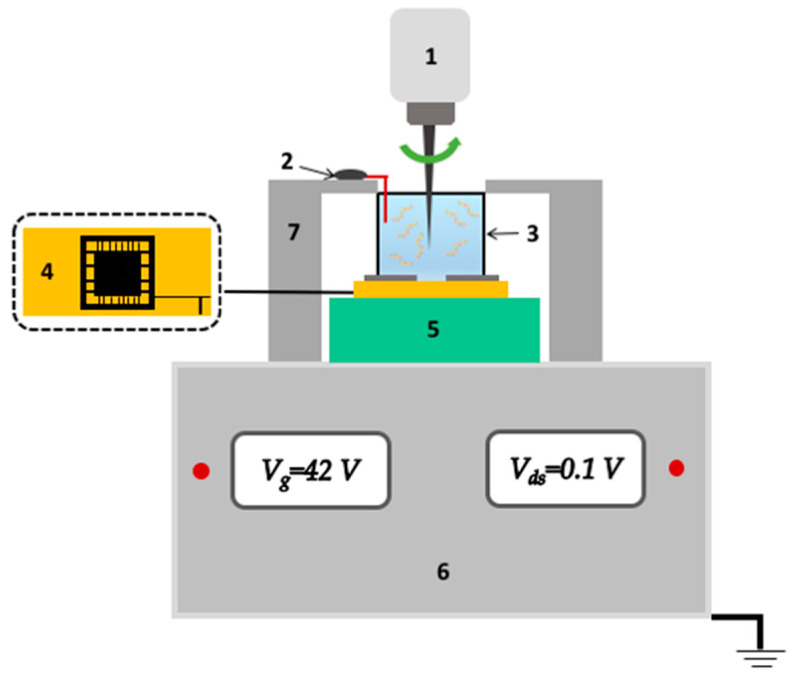
Schematic image of the analytical module of the SOI-NR biosensor. Numbers indicate the main components of the module: the stirrer (1), the platinum electrode (2), the measuring cell (3), the SOI-NR sensor chip (4), the chip holder (5), the ten-channel data acquisition and storage system (6), and the measuring cell holder (7).

The SOI-NR biosensor system consisted of analytical and electronic measurement modules (Figure 4). The main element of the analytical module was a chip bearing six pairs of SOI-NR structures (nanoribbons).

Prior to the measurements, the SOI-NR chip (Figure 4, (4)) was placed in the analytical module under the measuring cell (Figure 4, (3)), so that the chip surface served the cell bottom. The diameter of the chip’s sensitive area with SOI-NR structures was ~2 mm. The cell volume was 500 μL. The solution in the cell was stirred at 3000 rpm with a stirrer (Figure 4, (1)). During the experiment, the electronic measurement module provided simultaneous registration of the signal from 10 nanoribbons on the chip and its real-time visualization on the screen of the personal computer.

In order to improve the time stability of the biosensor operation, an additional platinum electrode (Figure 4, (2)) was immersed into the solution in the measuring cell [45].

### 2.9. Electrical Measurements

**Figure 5:**
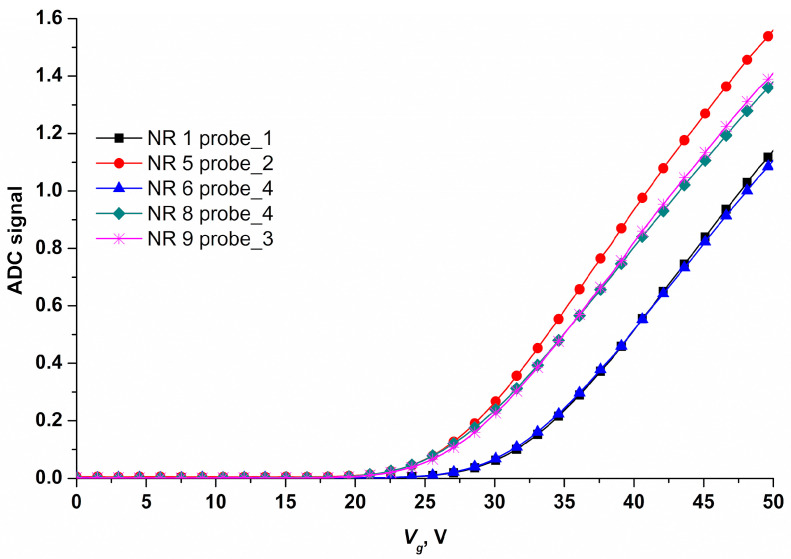
Typical drain-gate characteristics for five nanoribbon sensors of a SOI-NR chip recorded under the following conditions: 1 mM MCP, *V_g_* = 0 ÷ 50 V, and *V_ds_* = 0.1 V. The nanoribbons were sensitized with covalently immobilized oDNA probes: probe_1, probe_2, probe_3, or probe_4.

Electrical measurements, data acquisition, and analysis were performed using a ten-channel “Agama +” setup (Moscow, Russia). During the measurements, the nanoribbon surface was used as the transistor gate. The operating voltage for real-time experiments was determined based on the data of drain-gate characteristics (Figure 5).

The operating point of the sensor in the region of drain-gate characteristic (*I_ds_*(*V_g_*)) can be varied by applying voltage to the SOI-NR structure substrate. An exponential relation of the nanoribbon current to the surface potential is found for this point. In this way, the optimal operating voltage *V_g_* = 42 V was found to be optimal under the conditions of our experiments.

### 2.10. Biosensor Measurements

Biosensor measurements were performed in a buffer with low salt concentration (1 mM MCP) in order to avoid the Debye screening effect [39,49].

We used the chip sensitized with oDNA probes as described in Section 2.6. As the first step, we preformed the detection of model oDNAs (specified in Table 2) in purified buffer solution in order to determine the lowest oligonucleotide concentration detectable with our biosensor. In these experiments, a 150 μL volume of buffered oDNA solution was pipetted into the measuring cell containing 300 μL of 1 mM MCP. The oDNA concentration in the analyzed solution ranged between 10^−18^ M and 10^−15^ M. Solutions with different concentrations of four oDNAs, starting from the lowest one (10^−18^ M), were analyzed. After each analysis, the measuring cell was washed first with pure oDNA-free buffer, and then with ultrapure water (50 mL, 90 °C). 

As the second step, the detection of target miRNAs isolated from plasma samples was performed. The following protocol was used: a 7 μL volume of the solution of miRNAs, isolated from plasma of PC patients, was pipetted into the measuring cell containing 100 μL of 1 mM MCP (pH 7.4). The measurement protocol was identical to the one used in the first-step experiments with model oDNAs. Control experiments were performed under similar conditions, but with the solution of miRNAs isolated from the plasma of patients with left kidney cyst, while buffer from the protocol for miRNA isolation was used without biomolecules in order to detect the non-specific signal.

### 2.11. Data Analysis

The time function of the current was registered in real time. In order to account for non-specific interactions, values obtained in the blank experiment (i.e., in the experiment with purified oDNA-free buffer instead of oDNA solution) were used. These values were subtracted from absolute values obtained upon the analysis of the model oDNA solution. The registered changes in the current *I_ds_* through each nanoribbon were normalized to 1 by division by the initial current value: the ratio of *I_ds_* for a certain time period to the current value (*I_ds_*_0_) for the initial time period was calculated and expressed in relative units. After this, the difference between the normalized signal from working and control nanoribbons was measured (Section 2.6). The resulting time dependencies of the current (*I_ds_* (*t*)) were presented in the form of sensorgrams indicating the differential signal calculated by subtracting the signal received from the control nanoribbon from that received from the working nanoribbon.

## 3. Results

In our present research, the experiments were performed in two steps. The first step was the determination of the lower limit of oligonucleotide detection with the use of model oDNAs, which represented synthetic analogues of target miRNAs. The second step was the detection of miRNAs isolated from plasma samples of patients with confirmed PC diagnosis.

### 3.1. Determination of Method Sensitivity—Biospecific Detection of Target oDNAs in Buffer Solution

**Figure 6:**
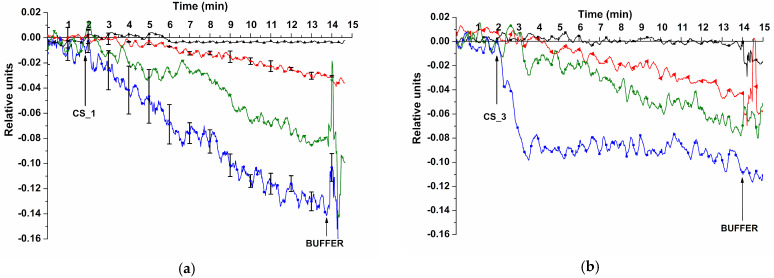
Typical sensorgrams obtained upon the detection of CS_1 (**a**) and CS_3 (**b**) model oDNAs with the SOI-NR biosensor. Experimental conditions: SOI-NR chip had n-type conductivity; nanoribbons were sensitized with oDNA probes (probe_1 (**a**) and probe_3 (**b**)); 1 mM MCP buffer; *V_g_* = 42 V; *V_ds_* = 0.1 V; total volume of solution in the measuring cell was 450 μL; concentrations of target oDNAs in the cell were 1.1 × 10^−18^ M (black curve), 1.1 × 10^−17^ M (red curve), 1.1 × 10^−16^ M (green curve), and 1.1 × 10^−15^ M (blue curve). Arrows indicate the time points of oDNA solution addition and of wash with pure oDNA-free buffer.

Figure 6 displays typical sensorgrams obtained upon the detection of model CS_1 and CS_3 oDNAs at concentrations ranging from 1.1 × 10^−18^ M to 10^−15^ M.

The sensorgrams shown in Figure 6 indicate that addition of model oDNA solutions to the final concentrations of either 10^−17^ M, 10^−16^ M, or 10^−15^ M led to an expected decrease in the conductivity of the nanoribbons. This decrease is explained by adsorption of negatively charged oDNA molecules onto the sensor surface. No signal was detected at the 10^−18^ M concentration of any of the oDNAs. The results were validated using standard deviation. Substitution of oDNA solution with pure buffer resulted in the same signal level. We explain this by slow dissociation of probe/CS complexes.

The lowest concentration of the model oDNAs, detectable in buffer with our SOI-NR biosensor, was 1.1 × 10^−17^ M for all model oDNAs tested (CS_1, CS_2, CS_3, and CS_4).

### 3.2. Biospecific Detection of miRNAs Isolated from Blood Plasma

**Figure 7:**
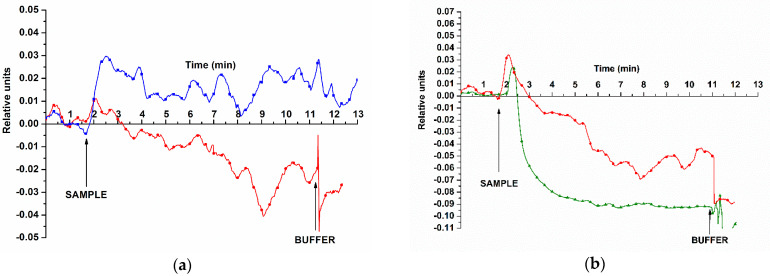
Typical sensorgrams obtained upon the detection of target miRNAs, isolated from plasma samples, using the SOI-NR biosensor. Experimental conditions: SOI-NR chip had n-type conductivity; nanoribbons were sensitized with oDNA probes (probe_1 (**a**); probe_2 (**b**)); the target miRNAs were isolated either from control plasma sample No. 27 (cyst of the left kidney, blue curve), or from plasma samples No. 44 and 5 (PC, red and green curves, respectively); 1 mM MCP; *V_g_* = 42 V; *V_ds_* = 0.1 V; total volume of solution in the measuring cell was 107 μL. Arrows indicate the time points of oDNA solution addition and of wash with pure buffer.

During the second step of our study, we successfully demonstrated the detection of miRNAs isolated from real plasma samples. Figure 7 displays typical sensorgrams obtained upon the detection of the target miRNAs.

The curves shown in Figure 7a indicate that addition of miRNAs, isolated from the plasma of the PC patient, resulted in decreased conductivity of nanoribbon sensors. The same trend was observed for blood plasma samples No. 5 and 44.

The signal recorded in control experiments changed insignificantly upon addition of miRNA samples isolated from plasma of patients with cyst of the left kidney (control sample No. 27; see Figure 7a, blue curve).

## 4. Discussion

Considering the methods of PC diagnosis, several points should be discussed. The first point is the sensitivity of the approaches employed. In many commercial clinical tests (for instance, in those studied by Murthy et al. [57]), the ELISA principle is typically employed. Their obvious disadvantage is insufficient specificity of the ELISA method. The lower limit of detection (LLD) attainable with the use of conventional ELISA-based assays is typically ~10^−12^ M [15]. At the same time, in order to provide early revelation of PC, the LLD of 10^−17^ M (or, at least, 10^−15^ M) is required [15]. Nanotechnology-based approaches to PC biomarker detection are believed to solve this problem [2], allowing one to overcome the LLD threshold. One of these approaches is based on the use of SOI-NR biosensors, which have very high sensitivity owing to the high surface-to-volume ratio of nanoribbons [48]. This is the key point in the detection of proteins [55] and nucleic acids [50] at ultra-low concentrations [36]. Therefore, these biosensors allow one to perform label-free real-time detection of target analytes with high selectivity, short response time, and good reproducibility of the results obtained [58,59,60,61]. 

Herein, in our experiments on the detection of model oDNAs, which represent synthetic analogues of target miRNAs, we have successfully demonstrated the highly sensitive and specific detection of target nucleic acid molecules. The novelty of the study comes from the use of the SOI-NR biosensor for the simultaneous detection of a palette of target miRNAs (miRNA-183 [31], miRNA-346 [51], miRNA-429 [52], and miRNA-484 [53]), which were reported to be associated with PC. The approach implemented has several key advantages. The first one is a very low (1.1 × 10^−17^ M) detection limit of the target nucleic acids. The second advantage is biospecificity of the detection provided by the sensitization of the nanoribbon surface with immobilized oligonucleotide molecular probes, which are complementary to the target PC-associated miRNAs. The third advantage is the applicability of our biosensor to the detection of the target miRNAs in real clinical plasma samples, as has been successfully demonstrated in our experiments. These advantages allow one to consider SOI-NR-based biosensors as promising tools for the early revelation and screening of PC in men.

Another point to be discussed is the type of biomarker used for the revelation of PC. Currently, the majority of both clinical [57] and laboratory [16,34,35,62] approaches to the PC revelation is based on the detection of prostate-specific antigen (PSA) [2]. Many approaches utilizing highly sensitive nanotechnology-based methods—such as those employing silicon nanowire electrical biosensors [41,63]—are also aimed at the detection of PSA [2]. Nevertheless, as is known to date, the PSA level does not inevitably indicate the presence of PC, and it is questionable whether one should solely rely on the results of PSA tests [7]. In parallel, Shen et al. emphasized that alterations in plasma levels of certain miRNAs can be used as predictors of PC aggressiveness [30]. In their review, Barani et al. [2] noted that the LLD, attainable for miRNAs with the use of nanotechnology-based [36,64] sensors, is considerably lower than that obtained for PSA with the use of nanoparticle-based approaches [34,35,38]. Thus, with respect to PC diagnosis and monitoring, miRNA markers represent promising alternative to PSA. As our study reported, we have demonstrated the successful applicability of SOI-NR nanotechnology-based biosensors for the highly sensitive simultaneous detection of a palette of PC-associated miRNAs.

## 5. Conclusions

The use of silicon nanoribbons as sensor elements in a biosensor system represents an innovative approach owing to their unique features. High surface-to-volume ratio of nanoribbons determines high sensitivity of detection of charged biomolecules of nucleic acids, providing their detection at ultra-low concentrations down to 1.1 × 10^−17^ M. 

Our study represents an advanced application of the SOI-NR biosensor for the detection of a palette of PC-associated miRNAs with high sensitivity and specificity. We have employed the SOI-NR biosensor with oDNA-sensitized sensor elements for highly sensitive label-free, real-time detection of prostate cancer-associated miRNAs, isolated from blood plasma samples. In contrast to antibodies, synthetic oDNA probes are cheap, chemically stable and durable, and their use further increases the feasibility and cost-effectiveness of the approach employed. The CMOS-compatible technology based on gas-phase reduction and lithography methods, which is suitable for mass production of chips containing dozens of nanoribbon structures, has been used for chip fabrication. These results form the basis for the development of advanced bioanalytical systems and diagnostic kits intended for early revelation of PC in men. Based on these results, future advances can include the integration of additional miRNA targets, multiplexed detection capabilities, and development of portable devices for point-of-care applications. The results of our study will be useful in the development of novel bioanalytical systems, which can further serve as the basis of diagnostic kits intended for early revelation of diseases in humans.

## Figures and Tables

**Table 1:** Nucleotide sequences of oDNA probes immobilized on the surface of nanoribbons.

oDNA Probe Name	oDNA Probe Sequence
probe_1	5′-(NH_2_)-(T)_10_TCGTGGATCTGTCTCTGCTCTGTTTATGGCCCTTCGGTAATTCACTGACTGAGACTGTTCACAGTGAATTCTACCAGTGCCATACACAGAACAGGAGTCACACTGCGG
probe_2	5′-(NH_2_)-(T)_10_CCGCTCTGCCCAGGCAGCTGCAGGCCCAGCCCCTGCCTCCTTCAGAGCAACAGAGAGGCAGGCATGCGGGCAGACAGACGCCCAACACAGAGACC
probe_3	5′-(NH_2_)-(T)_10_GCAGCGGATGGACGGTTTTACCAGACAGTATTAGACAGAGGGCCAGGTCTAACCATGTCTGGTAAGACGCCCATCGGCCGGCG
probe_4	5′-(NH_2_)-(T)_10_CGCCAAAAAAGCCAGGGTCACCCCCCGGGAAAGTCCCTATTTAGGGGTTTATCGGGAGGGGACTGAGCCTGACGAGGCT

**Table 2:** Nucleotide sequences of model oDNAs, which represent synthetic analogues of the respective target miRNAs.

Model oDNA Name	Model oDNA Probe Sequence	Respective Target miRNA Name	Ref.
CS_1	CCGCAGAGTGTGACTCCTGTTCTGTGTATGGCACTGGTAGAATTCACTGTGAACAGTCTCAGTCAGTGAATTACCGAAGGGCCATAAACAGAGCAGAGACAGATCCACGA	hsa-mir-183	[31]
CS_2	GGTCTCTGTGTTGGGCGTCTGTCTGCCCGCATGCCTGCCTCTCTGTTGCTCTGAAGGAGGCAGGGGCTGGGCCTGCAGCTGCCTGGGCAGAGCGG	hsa-mir-346	[51]
CS_3	CGCCGGCCGATGGGCGTCTTACCAGACATGGTTAGACCTGGCC CTCTGTCTAATACTGTCTGGTAAAACCGTCCATCCGCTGC	hsa-mir-429	[52]
CS_4	AGCCTCGTCAGGCTCAGTCCCCTCCCGATAAACCCCTAAATAGGGACTTTCCCGGGGGGTGACCCTGGCTTTTTTGGCG	hsa-mir-484	[53]

**Table 3:** The characteristics of blood plasma samples.

	Sample	Age	Gender	Diagnosis	TNM Stage	Total Gleason Score (Points)
PC samples	Sample No. 44	68	male	prostate cancer	T1cN0M0	6
	Sample No. 5	59	male	prostate cancer	T2cN0M0	6
Control	Sample No. 27	51	male	cyst of the left kidney	–	–

## Data Availability

Correspondence and requests for materials should be addressed to K.V.G.
